# Deciphering the Formulation Secret Underlying Chinese Huo-Clearing Herbal Drink

**DOI:** 10.3389/fphar.2021.654699

**Published:** 2021-04-22

**Authors:** Jianan Wang, Bo Zhou, Xiangdong Hu, Shuang Dong, Ming Hong, Jun Wang, Jian Chen, Jiuliang Zhang, Qiyun Zhang, Xiaohua Li, Alexander N. Shikov, Sheng Hu, Xuebo Hu

**Affiliations:** ^1^ Laboratory of Drug Discovery and Molecular Engineering, Department of Medicinal Plants, College of Plant Science and Technology, Huazhong Agricultural University, Wuhan, China; ^2^ National and Local Joint Engineering Research Center (Hubei) for Medicinal Plant Breeding and Cultivation, Wuhan, China; ^3^ Hubei Provincial Engineering Research Center for Medicinal Plants, Wuhan, China; ^4^ Hubei Cancer Hospital, Tongji Medical College, Huazhong University of Science and Technology, Wuhan, China; ^5^ College of Food Science and Technology, Huazhong Agricultural University, Wuhan, China; ^6^ Department of Technology of pharmaceutical formulations, Saint-Petersburg State Chemical Pharmaceutical University, Saint-Petersburg, Russia

**Keywords:** ICAM-1, traditional Chinese medicine, Shang Huo, inflammation, herbal tea, herbal drink

## Abstract

Herbal teas or herbal drinks are traditional beverages that are prevalent in many cultures around the world. In Traditional Chinese Medicine, an herbal drink infused with different types of medicinal plants is believed to reduce the ‘Shang Huo’, or excessive body heat, a status of sub-optimal health. Although it is widely accepted and has a very large market, the underlying science for herbal drinks remains elusive. By studying a group of herbs for drinks, including ‘Gan’ (*Glycyrrhiza uralensis* Fisch. Ex DC.), ‘Ju’ (*Dendranthema morifolium* (Ramat.) Tzvelev), ‘Bu’ (*Microcos paniculata* L.), ‘Jin’ (*Lonicera japonica* Thunb.), ‘Xia’ (*Prunella vulgaris* L.), and ‘Ji’ (*Plumeria rubra* L.), the long-term jargon is connected with the inflammation of modern immunology through a few pro-inflammatory markers. *In vitro* studies have indicated that cellular inflammation is lowered by Ju and Jin either individually or synergistically with Gan. Among all herbs, only Gan detoxicated cellular toxicity of Bu in a dose dependent manner. The synergistic formulation of Ju and Gan, or Jin and Gan, in a reduction of Shang Huo, was tested *in vivo*. Both combinations exhibited a lower percentage of neutrophils, monocytes, and CD4^+^/CD8^+^ ratio in the blood, as well as inflammatory cytokines. Furthermore, body weight in the combinatory groups was more stable than treatments using single herbs. The combination of old traditional oriental methods with Western science logistics, has resulted in the formulation of different herbs into one concoction for the use of detoxification and synergism.

## Introduction

A great variety of herbal drinks are appreciated across the world. Besides the well-studied green tea which is used for the mitigation of oxidative stress (Serafini et al., 2011), many other kinds of herbs are also used as beverages. For example, barley (*Hordeum vulgare* L.) in East Asia (Tatsu et al., 2020), rooibos tea [*Aspalathus linearis* (Burm.f.) Dahlg] in South Africa (van Heerden and Hardie, 2020), lemongrass [*Cymbopogon citratus* (DC.) Stapf] in India and Pakistan (Kieling and Prudencio, 2019), thyme (*Thymus vulgaris* L.) in Germany (Kulisić et al., 2007), badan [*Bergenia crassifolia* (L.) Fritsch] and Ivan-tea (*Epilobium angustifolium* L.) in Russia (Shikov et al., 2014; Shikov et al., 2017), and yerba mate (*Ilex paraguariensis* A.St.-Hil.) in South America (Bracesco et al., 2011). Similarly, herbal drinks in Traditional Chinese Medicine (TCM) are used to treat slight ailments. Different from other types of TCM, which are more potent in treating substantial diseases, herbal drinks in TCM are milder, consumed in smaller doses, but is stronger, clearly differentiated from common teas. In TCM theory it is believed that an herbal drink can remove the over-heating, or “Shang-huo” of the body. Over-heating of the body is one of the five culprits incurring disease, based on the oldest TCM transcript “Huang-di Nei-jin,” which is over 2000 years old. The concept has been passed down to TCM practitioners ever since. The symptoms of TCM Shang-huo are commonly represented by a sore throat, mouth sores, swollen gums, red eyes, irritability, insomnia, and dry stool (Liu et al., 2016; Xie et al., 2017). Along with the increase in urbanization and industrialization, the rapid pace of living and a high-pressure society have placed more and more people in extended periods of anxiety, tension, insomnia, and melancholy. These signs are characteristic of a state of decreased resistance to stress, which could be ameliorated by adaptogens (Panossian et al., 2021). These symptoms of suboptimal health are also commonly associated with the above Shang-huo indicators of TCM, which are actually similar to the phenomena of topical inflammation in immunology (Pan et al., 2020). In accordance with the observation, many studies have indicated that herbs used in herbal drinks present a certain anti-inflammatory activity (Wang et al., 2008; Huang et al., 2012).

On the other hand, such herbal drinks are usually formulated by a few types of herbal extracts. There is a general formulation principle for all TCM based on another important transcript “Shen-nong Ben-cao Jing” (SBJ), which is contemporary of the “Huang-di Nei-jin.” The SBJ states that an ideal formula should contain a group of harmonious herbs, each playing unique roles, mimicking a well-organized society with a monarch, a minister, an assistant, and a guide (Serafini et al., 2011). However, the principle of SBJ is hard to follow in practice because of distinct rules for its formulation, even when treating a disease with clear symptoms, let alone diseases like Shang-huo with obscure manifestations. Many TCM formulas have therefore been passed onto descendants in secret.

In the current study, Shang-huo, which is linked to inflammation through an inflammatory endothelial cell model was studied. A collection of six herbal materials were investigated in this study: *Glycyrrhiza uralensis* Fisch. Ex DC. (Leguminosae), *Dendranthema morifolium* (Ramat.) Tzvelev (Asteraceae), *Microcos paniculata* L. (Malvaceae), *Lonicera japonica* Thunb. (Caprifoliaceae), *Prunella vulgaris* L. (Lamiaceae), and *Plumeria rubra* L. (Apocynaceae). Each herb’s ability to reduce Shang-Huo is indicated and then quantified by inflammatory protein markers. With the quantification, each herb’s contribution to reducing the Shang-Huo was compared in parallel. A combination of different herbs in one pot also showed synergistic efficacy. Some herbs were not beneficial to reducing Shang-huo, but it did alleviate cytotoxicity. The findings in cells were also verified *in vivo*. Mice studies also showed that a combination of different herbs is favorable for maintaining immune biochemical profiles and keeping a steady body weight while ingesting a single type of herb led to weight loss.

## Materials and Methods

### Herbal Extraction

**FIGURE 1:**
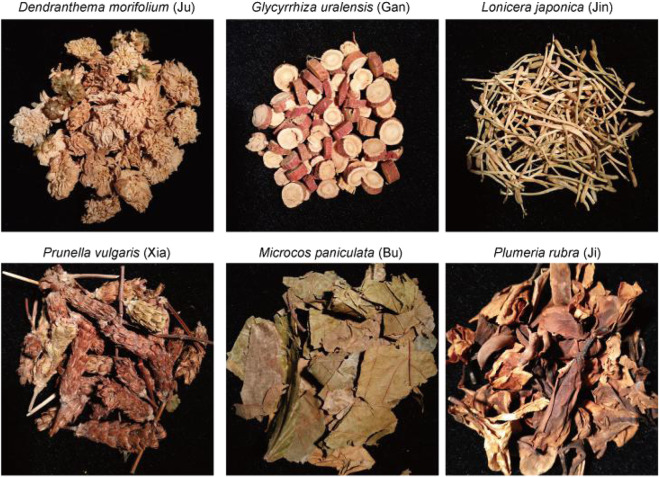
Photos of medicinal materials of 6 herbs used in this study. These are flowers of Ju, Jin and Ji, roots of Gan, Leaves of Bu and ripe flowers and fruits of Xia. All materials were used as dry samples.

The herbal materials investigated in this study consisted of six herbs: “Gan” (the root of *Glycyrrhiza uralensis* Fisch. Ex DC.), “Ju” (The flower of *Dendranthema morifolium* (Ramat.) Tzvelev), “Bu” (The leaf of *Microcos paniculata* L.), “Jin” (The flower of *Lonicera japonica* Thunb.), “Xia” (The fruit body of *Prunella vulgaris* L.), and “Ji” (The flower of *Plumeria rubra* L.) (Figure 1). All herbs used in this study were of Chinese origin and inspected to confirm their identity by a certified TCM pharmacist (Prof. Mo Wang). The quality of the materials was verified by high-performance liquid chromatography (HPLC; Supplementary Figure S1; Supplementary Table S1). Each herbal material was soaked in 1.5 L of water for 30 min at room temperature (RT) and then boiled for 2 h, resulting in a volume of 1 L. After filtration, concentration, and lyophilization, the powder was stored at −20°C until use. For Gan and Ju, 20 g of each material was used, and the resultant powder was 6.5 and 3.3 g, respectively. For Ju, Jin, Ji, and Xia, the initial material was 10 g from each, and the resultant powder for further experimentation was 4.57, 3.39, 2.68, and 2.01 g, respectively.

### Mammalian Cell Culture

Human microvascular endothelial cells (HMEC-1) were gifted from Dr. Moonsoo M Jin (Weill Cornell Medicine). HMEC-1 cells were cultured in MCDB 131 medium (Sigma) supplemented with 10% FBS (Natocor—Industria Biológica, Argentina), 1 mg/ml hydrocortisone (Sangon Biotech, Shaihai, China), and 10 ng/ml recombinant human epidermal growth factor (Sangon Biotech, Shaihai, China). For induction of inflammation, HMEC-1 cells were treated with 1 mg/ml of LPS 3 h after the treatment with herbal extracts. The mammalian cells were maintained at 37°C in a 5% CO_2_ humidity incubator.

### MTT Assay

To determine cell viability, the colorimetric MTT assay was used. HMEC-1 cells (1 × 10^4^ cells/well) were cultured in MCDB131 media in a 96-well plate at 37°C and exposed to herbal extract with different concentrations for 24 h. Cells treated with medium concentration were only used as a negative control. After removing the supernatant of each well and followed by PBS washing, cells were added with 20 µL of MTT solution (5 mg/ml in PBS) and 80 µL culture medium. The cells were then incubated for another 4 h until the formazan crystals were dissolved in dimethyl sulfoxide (100 µL). The absorbance intensity was measured by a microplate reader (Bio-RAD 680, United States) at 570 nm with a reference wavelength of 630 nm. All experiments were performed in triplicate, and the relative cell viability (%) was calculated as a percentage relative to the untreated control.

### CCK-8 Assay

To determine cell viability under a different treatment, the colorimetric CCK-8 metabolic activity assay was used. HMEC-1 cells (1 × 10^4^ cells/well) were cultured in MCDB131 media in a 96-well plate at 37°C, and then exposed to extracts with different concentrations for 24 h. Cells treated with a medium concentration only served as a negative control. After removing the supernatant of each well, PBS washing was done, before adding cells with 10 µL of CCK-8 solution (5 mg/ml in PBS) and 90 µL of culture medium. The cells were then incubated for another 4 h, and the absorbance intensity was measured by a microplate reader (Bio-RAD 680, United States) at 450 nm. All experiments were performed in triplicate, and the relative cell viability (%) was calculated as a percentage relative to the untreated control.

### Immunofluorescence Flow Cytometry

Immunofluorescence flow cytometry for ICAM-1 analysis on the HMEC-1 surface was conducted according to the method described previously (Kang et al., 2011). Flow cytometry for IL-8 and MCP-1 analysis followed the instructions of the Multi-Analyte Flow Assay Kit (LEGENDplex^TM^, Biolegend, United States). Mice peripheral blood was treated with lysis buffer (1.5 M NH_4_Cl, 10 nM NaHCO_3_, 1 mM EDTA·2Na) and washed by PBS. The cells were then permeabilized and stained with fluorescent-conjugated monoclonal antibodies to CD3, CD4, CD8, CD14, CD11b, and Ly-6G/Ly-6C (BD Biosciences, United States). After washing, the cells were analyzed on a FACSCalibur (Guava easyCyte, Millipore).

### RNA Extraction and Semiquantitative RT-PCR

RNA extraction was conducted according to a previous method (Kang et al., 2011). Then 500 μg of total RNA was reverse-transcribed at 42°C for 20 min, followed by 85°C for 5 s using a reverse transcription kit (TRUEscript 1st Strand cDNA, Aidlab Biotechnologies, China) in a thermal cycler (Thermal Cycler A100, LongGene Scientific Instruments, China). The cDNA product was used for real-time gene amplification analysis. Master mix 2X qPCR kit (2x Sybr Green qPCR Mix, Aidlab Biotechnologies) was used to amplify the specified genes for quantitative PCR. Intron spanning primers were designed from the National Institute of Health qPrimerDepot using accession codes NM_000201 (ICAM-1), NM_001078 (VCAM-1), NM_002982 (MCP-1), NM_000634 (IL-8), and NM_002046 (GAPDH).

### Animal Study

The animal experiments were conducted according to the guidelines for the care of animals of Huazhong Agricultural University (Wuhan, China), and were approved by the Institutional Animal Care and Ethics Committee (HZAUMO-2018-057). C57BL/6 mice (female, 6 weeks old) was purchased from the Hubei Provincial Center for disease Control and Prevention and maintained in the animal facility at Huazhong Agriculture University (Wuhan, China). Standard guidelines for laboratory animal care were followed. The study protocol was approved by the Huazhong Agricultural University Animal Care Committee. The mice were randomly divided into different groups as indicated, each with six to eight mice. Lyophilized herbal extracts of different amounts were resuspended in saline buffer and orally administered to mice (200 μL) twice daily for 4 days. On day 4, 3 h after the administration of the herbal extracts, the mice were intraperitoneally injected with 10 mg/kg lipopolysaccharide (LPS; *Escherichia coli*. 026B6, Sigma, United States) in 300 μL saline buffer. The control was administered the same volume of saline buffer. The body weight of the mice was recorded every 24 h for 10 days.

For lymphocytes analysis, the peripheral blood was obtained by orbital sinus bleeding. The blood samples were centrifuged at 3,000 rpm for 5 min in an anticoagulant tube. The supernatant was then discarded, and red blood cell lysis buffer (Biolegend) was added to disrupt red blood cells. After sitting on a bench for 10 min, the sample was centrifuged, and the pellet was washed again by saline buffer. The pelleted lymphocytes were examined by a cytometer (Guava easyCyte, Millipore).

### Statistical Analysis

**FIGURE 2:**
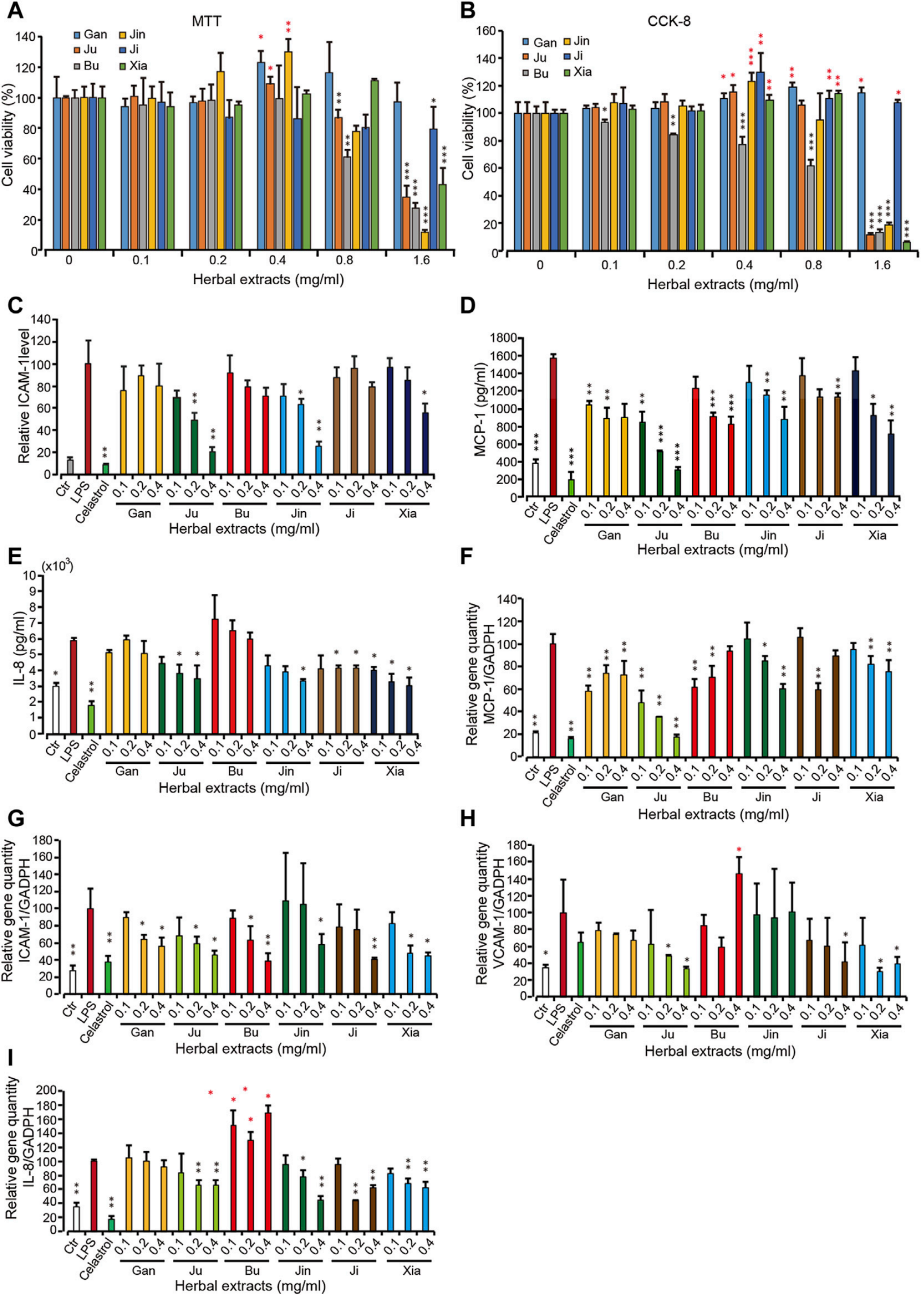
The cellular viability and pro-inflammatory protein and gene level upon treatment with 6 herbal extracts of different concentration in inflamed HMEC-1 cells **(A,B)** The assay was done by MTT or CCK-8. **(C–E)** Pro-inflammatory protein expression under treatment of herbal extracts. HMEC-1 cells were treated with different herbal extracts at indicated concentration for 6 h and then LPS was added for 3 h. Cells were then retrieved for ICAM-1 **(C)** and supernatant was used for MCP-1 **(D)** and IL-8 **(E)** quantification. **(F–I)** The quantitative PCR was conducted for gene MCP-1 **(F)**, ICAM-1 **(G)**, VCAM-1 **(H)** and IL-8 **(I)**. The relative gene quantity was normalized with GADPH and cells without any treatment were used as blank control (Ctr). Error bars represent standard deviation, *n* = 3. Single, double or triple asterisks represent *p* ≤ 0.05, *p* ≤ 0.01 and *p* ≤ 0.001, respectively (student’s *t*-test).

**FIGURE 3:**
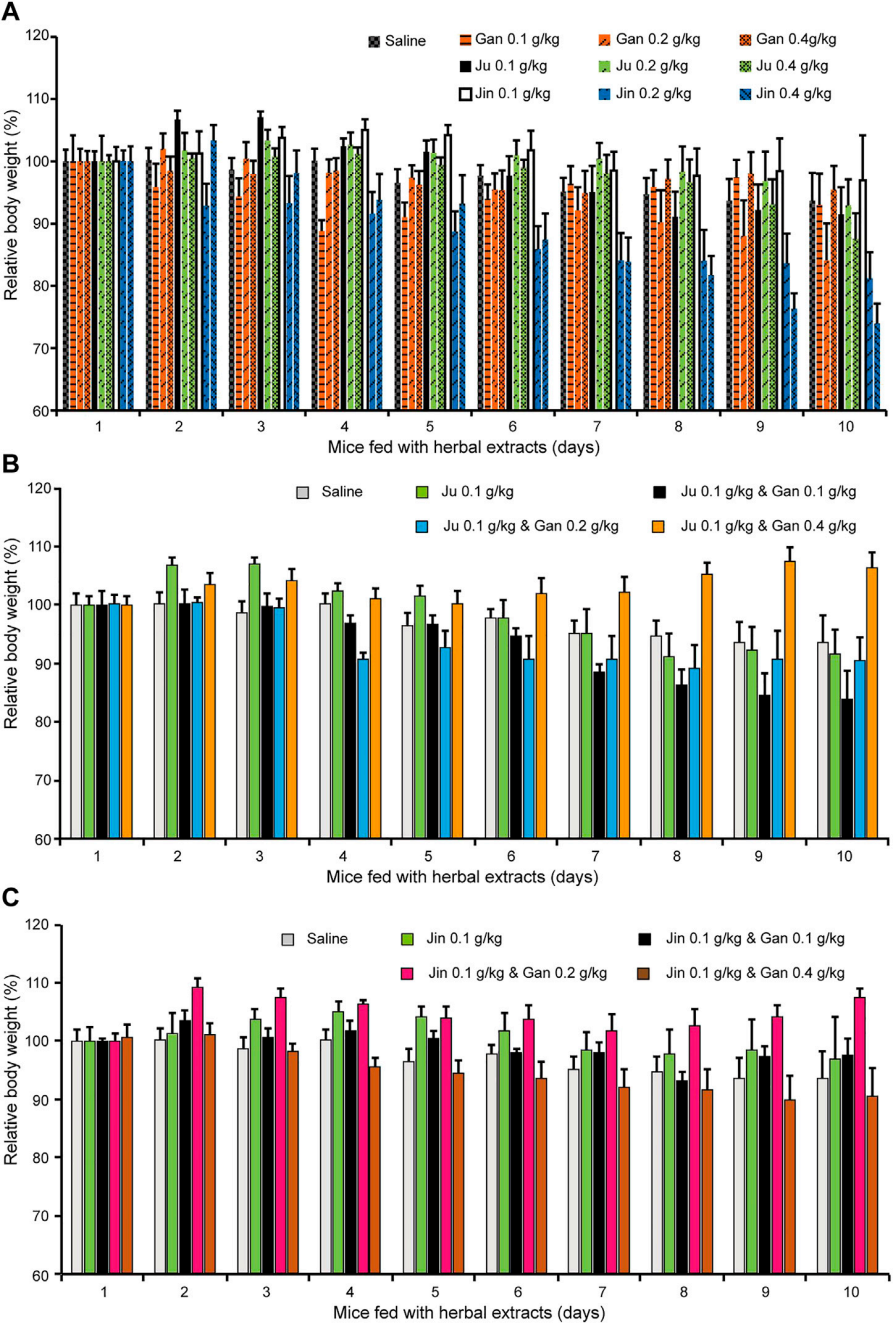
The body weight changed upon oral administration of herbal drink. Mice fed with different combination of herbal drink for 10 days and the body weight of each day was recorded. **(A)** Single herb extract with different concentration was administrated. **(B)** Ju and Gan were co-administrated with different concentration. **(C)** Jin and Gan were co-administrated with different concentration.

**FIGURE 4:**
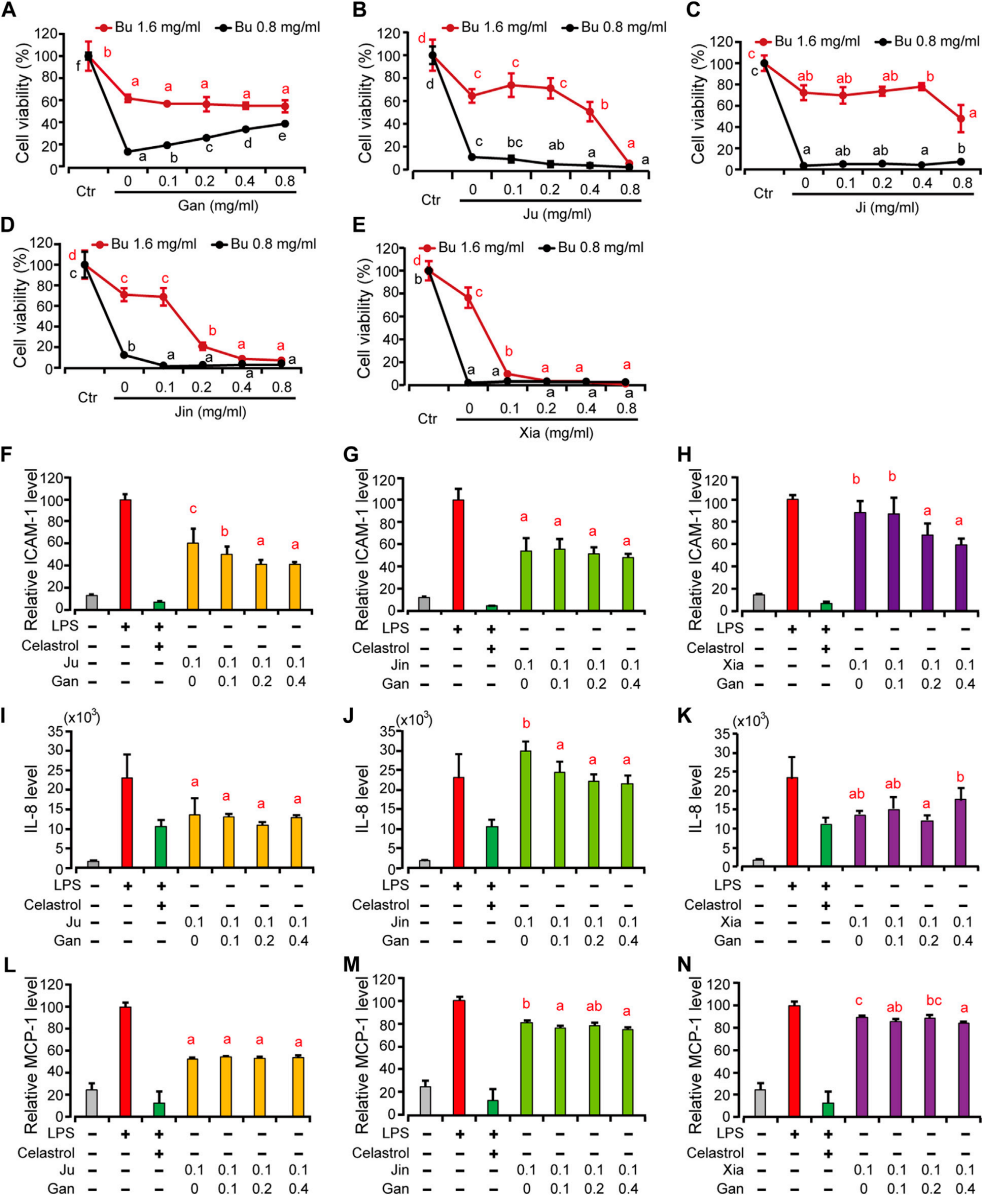
The HMEC-1 viability and pro-inflammatory protein expression treated by different combination of herbal drink treated by Bu and other herbal extracts. **(A–E)** Gan, Ju, Ji, Jin and Xia at different concentration were combined with Bu to treat HMEC-1 cells and the cellular viability was determined with a blank control (Ctr) as 100% **(F–H)**, HMEC-1 cells were treated with different herbal extracts at indicated concentration for 6 h. And then LPS was added for 3 h. Cells were then retrieved for ICAM-1 **(F–G)** and supernatant for MCP-1 **(I–K)** and IL-8 **(L–N)** quantification. Error bars represent standard deviation, *n* = 3. Different letter above the line or bars indicates significant pair-wize differences in LSD post-hoc test (*p* < 0.05).

**FIGURE 5:**
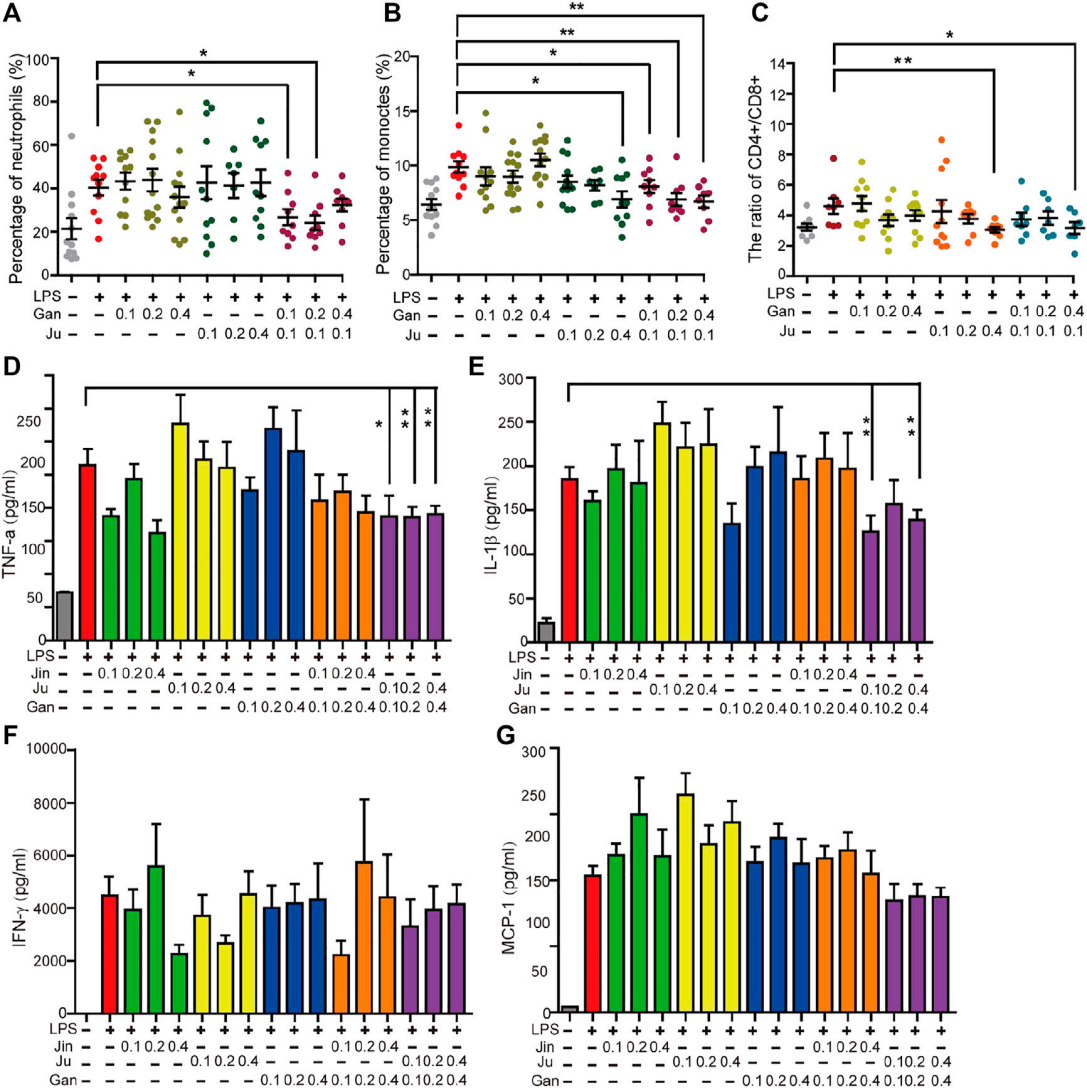
Oral administration of herbal drink changed the mice blood white cells ratio, cytokines and the body weight. The percentage of neutrophils **(A)**, monocytes **(B)** and the ratio of CD4^+^ and CD8^+^ T lymphocytes **(C)** in peripheral blood were measured. Cytokines in peripheral blood cells including TNF-α, IL-1β, MCP-1, and IFN-γ were measured **(D–G)**. Single or double asterisks represent p ≤ 0.05 and p ≤ 0.01, respectively (student’s t-test).

Data was expressed as mean ± standard deviation (SD) of at least four identical samples. The statistical analysis of the data was carried out using GraphPad Prism 7. The unpaired student’s t-test was used to determine statistical significance in comparison to the matched controls (Figures 2, 3). One-way ANOVA was used to compare mean responses among different treatments and the control (Figures 4, 5), followed by a Tukey’s post-hoc test to determine statistical significance.

## Results

### Quantification of Herbal Shang-Huo Reducing Capability in an Endothelial Cell Model

To explore the underlying rationale of herbal drinks, we selected a group of popular herbs, including licorice (*Glycyrrhiza uralensis* Fisch.) or “Gancao” in Chinese (“Gan” for short, hereafter used), chrysanthemum, “Juhua” or “Ju” (*Chrysanthemum morifolium* Ramat.), honeysuckle, “Jinyinhua” or “Jin” (*Lonicera japonica* Thunb.), frangipani, “Jidanhua” or “Ji” (*Plumeria rubra* L.), shiral, “Buzhaye” or “Bu” (*Microcos paniculata* L.), and self-heal, “Xiakucao” or “Xia” (*Prunella vulgaris* L.), to test their effectiveness against Shang-huo (Figure 1). The acronym was chosen according to Chinese phonetic pronunciation. These six herbs were individually extracted by hot water infusion. Referring to The Chinese *Pharmacopoeia* (version 2015), one to two chemicals from each herb were quantified by HPLC for quality control and standardization (Supplementary Figure S1; Supplementary Table S1).

On top of our previous cellular model, lipopolysaccharides (LPS)-induced human microvascular endothelial cells (HMEC-1), an inflammatory cell mode was applied as the cellular model (Kang et al., 2011; Zhang et al., 2018) to test the compatibility and effectiveness of Shang Huo to anti-inflammation. To select an appropriate concentration for the study, the cytotoxicity was assayed for each of these herbal extracts in HMEC-1 cells. It showed that Gan and Ji had no cytotoxicity to the cells, and even promoted the cell viability at all tested concentrations. However, the rest of the herbs all exhibited cytotoxicity at higher concentrations (Figures 2A,B), with Bu being the only one showing concentration-dependent toxicity at all concentrations (Figures 2A,B). Then HMEC-1 cells were treated with these herbs in a concentration range without cytotoxicity. The protein expression level of a few pro-inflammatory factors, including intercellular adhesion molecule 1 (ICAM-1) on the endothelia surface and chemokines monocyte chemotactic protein (MCP)-1 and interleukin (IL)-8, were investigated. As expected, all three cytokines were greatly induced by LPS. In the presence of LPS, Ju, Bu, Jin, and Xia all imposed a concentration-dependent ICAM-1 reduction, while Gan and Ji did not lower the ICAM-1 level significantly (Figure 2C). Another cell surface inflammatory marker, VCAM-1, was barely detected in all treatments (data not shown). The level of a group of cytokines including TNF-α, IL-1β, MCP-1 and IFN-γ, IL-8 and MCP-1 was examined. Because HMEC-1 is not an immune cell, we could only detect IL-8 and MCP-1 expression, which showed a similar trend with ICAM-1 (Figures 2D,E). Overall, Ju and Jin reduced the level of these pro-inflammatory factors, while Gan and Bu had the least effects. Correspondingly, the mRNA level of these genes was also reduced when the cells were treated by these herbal extracts (Figures 2F–I).

### The Synergistic Effects of Herbal Drink Formulation

We then focused on interpreting the rationality of mixing different herbs into a one-pot drink, since the anti-inflammatory strength of each herb is different. Because Bu exhibited a concentration-dependent toxicity, we tested the cellular viability with a combination of Bu and the other five herbal extracts, respectively. It showed that only Gan exhibited a concentration-dependent rescue of the cellular viability (Figures 4A–E). Gan is called the “prime minister” of all TCM because it is a core component in most TCM formulas. It is believed to possess the unique ability of harmonizing and neutralizing the rest of the components in formulas (Kao et al., 2014).

Besides neutralizing the toxicity of other herbs at higher concentrations, we wonder if Gan also contributes to the efficacy if the concentration of the herbal drink is lower. To further clarify the difference that Gan makes in an herbal extract, we selected Ju, Jin, and Xia, which all have a cytotoxicity over 0.1 mg/ml (Figure 2). The result showed that, the higher the concentration of Gan, the lower the ICAM-1 expression on the surface of HMEC-1 cells (Figures 4F–H). The group of 0.1 mg/ml Gan and 0.1 mg/ml Ju significantly suppressed ICAM-1 expression, compared to 0.1 mg/ml extract of Ju only. The same result was achieved when 0.2 mg/ml Gan and 0.1 mg/ml Xia as a group was tested against 0.1 mg/ml Xia alone. In terms of ICAM-1 reduction, the synergistic effect of Jin and Gan were marginal. Jin, Ju, or Xia separately lowered the expression of IL-8 and MCP-1. Because Gan detoxicated other herbs, we tested the combination of Gan with Jin, Xia, and Ju. It showed that Jin and Gan, or Xia and Gan, but not Ju and Gan, exhibited significant synergism (Figure 4).

### Quantification of Herbal Shang-Huo Reducing Capability *In Vivo*

The anti-inflammation herbal extracts were tested in a mouse model. Systemic inflammation in C57BL/6 mice was induced by LPS with intraperitoneal injection. The quantity of neutrophils and monocytes are subjected to inflammatory stimulation (Hajishengallis and Hajishengallis, 2014; Yang et al., 2014), and thus they represent a status of cellular inflammation. The percentage of neutrophils and monocytes in the peripheral blood was investigated in all treatments. This level was significantly increased by LPS treatment (Figures 5A,B). For herbal drinks, all treatments were orally fed three days before LPS. Individual Gan or Ju did not notably alter the percentage of neutrophils. However, the combination of both herbs significantly reduced the level (Figure 5A). A similar result was obtained for the percentage of monocytes (Figure 5B). The stabilization of the CD4^+^ to CD8^+^ T cells was investigated during the treatment. T cells play a major role in cell-mediated immunity. The ratio of CD4^+^ to CD8^+^ cells is a marker of HIV progression since it accurately indicates the overall immunity dysfunction imposed by the virus (Mcbride and Striker, 2017). Furthermore, the ratio of CD4^+^ to CD8^+^ cells increases in inflammatory diseases (Watkins et al., 2013). The subsets of T lymphocytes were analyzed during herbal extract treatment. Upon LPS induction, the ratio of CD4^+^/CD8^+^ T lymphocytes in the peripheral blood of mice significantly increased compared to the saline group (Figure 5C). A single Gan or Ju treatment decreased the ratio of CD4^+^/CD8^+^. When the concentration of the herbal drink went higher, the inhibitory effect was strengthened, however, without statistical significance (Figure 5C). The combination of Gan and Ju also lowered the ratio, but there was only a significant difference if the Gan concentration was high (Figure 5C).

We investigated a few classical cytokines including TNF-α, IL-1β, MCP-1, and IFN-γ in the serum of mouse peripherical blood. Each of these cytokines was significantly increased by LPS treatment. However, surprisingly, herbal treatment of Jin, Ju, or Gan alone enhanced the level of these cytokines (Figures 5D–G). The levels of cytokines were back to normal when Jin or Ju was composed with Gan, indicating that a combination of two herbs helps reduce the inflammatory level.

Mouse body weight was recorded during the treatment (Figures 3A–C). The body weight of mice fed Gan or Ju alone had no obvious change. However, it was clearly shown that Jin induced a dose-dependent body weight reduction (Figure 3A). On the other hand, Gan greatly enhanced the body weight when combined with either Ju or Jin (Figures 3B,C). However, the most beneficial dosage for each combination was different. Gan assisted Ju (0.1 g/kg) at 0.4 g/kg, but for Jin, it was 0.2 g/kg.

## Discussion

Traditional medicine plays an essential role in our life. However, its scientific foundation has always been viewed with skepticism since modern science has dominated biomedical study and practice (Parker, 2003). Facing the strict paradigm of conventional science, the seemingly ambiguous and vague traditional medicine is as a compromise usually called alternative or complementary medicine. However, traditional medicine could be demystified using a modern approach (Gu and Pei, 2017; Li et al., 2018). Herbal drinks are a mild treatment for slight ailments among all means of TCM. This study lent a concept of inflammation from immunology. An abstruse jargon “Shang Huo” could be directly correlated to inflammation by quantitative measurement of inflammation related proteins. This quantification assists in comparing the capability of inhibiting excessive “Huo” between different herbs, which was impossible to differentiate using classic theories of TCM. In a previous study, the endothelial cells HMEC-1 were set up to screen anti-inflammatory chemicals from a library. ICAM-1 was chosen as a marker for the quantitative detection of its ligand and lymphocyte function-associated antigen 1 (LFA-1). LFA-1 is an integrin that is activated and specifically bound to ICAM-1 in inflammation signaling (Luo et al., 2007). With LPS as the positive control, the anti-inflammatory strength of each compound could be quantified (Zhang et al., 2018). The best inflammation-inducing or promoting chemicals were screened by this platform (Zhang et al., 2018). In the current study the system was extended to compare the Huo-clearing capability of each herb, since we deduced that inflammation could be a counterpart of “Huo” in TCM.

**FIGURE 6:**
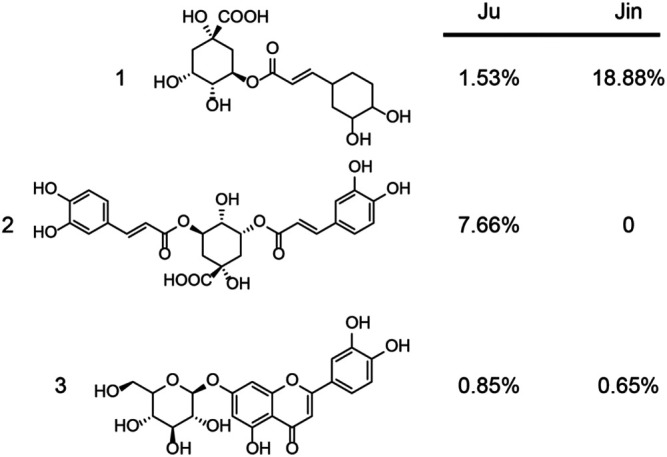
The content of major bioactive compounds from Ju and Jin. The major compounds **1** (chlorogenic acid), **2** (3,5-O-dicaffeoylquinic acid), **3** (luteolin-7-O-glucoside) and their content (w/w) was measured.

Similar to single chemicals, the capability of Shang Huo clearing herbs were also identified and ranked using this method. Most of the herbs tested in this study are used in TCM to clear Huo (Tatsu et al., 2020). However, there was no direct tool to compare their capability. The method allowed us to screen the best ones from a pool of candidates. Although Ju, Jin, Xia, Bu, and Ji are widely used as herbal tea ingredients to clear Huo, their actual potency has not been quantitatively compared in parallel. In this study, we could clearly see that Ju and Jin take the lead over others (Figures 2, 5). Interestingly, two of three major bioactive compounds of Ju and Jin are identical (Figure 6). Furthermore, content **1** (chlorogenic acid) and **3** (luteolin-7-O-glucoside) in Ju was less than Jin; while a relatively high content of compound **2** (3,5-O-dicaffeoylquinic acid) was not found in Jin (Figure 6). Although the contribution of the anti-inflammatory effect of these compounds was not individually characterized, Ju exhibited better anti-inflammation than Jin (Figures 2, 5). It indicated that two might be a key factor for the better anti-inflammatory effect of Ju than Jin.

Gan is very particular in TCM because it is used in most herbal formulas. Thus, Gan is called the prime minister of TCM (Kao et al., 2014). Glycyrrhizae radices are used in Russian traditional and officinal medicine as n expectorant and emollient, which is related to anti-inflammatory properties (Shikov et al., 2021). Anti-inflammatory features of licorice are utilized in Indian systems of traditional medicine (Pastorino et al., 2018). The philosophy of TCM believes that Gan harmonizes all herbs in most formulas (Hosseinzadeh and Nassiri-Asl, 2015). In this study, Gan showed a mild anti-inflammatory effect (Figure 2). However, the importance of Gan was further verified when it neutralized the toxicity of an herb, while other herbs in the group were unable to exhibit similar modes (Figure 2). Gan also further increased the anti-inflammation effect (Figure 4).

In an effort to explore the formulation principle, the mouse study was conducted. The percentage of neutrophils, monocytes, and CD4/CD8 ratio are all related to inflammation. To check the immune cellular change, mice were fed Gan, Ju, or both. The results clearly showed that Gan or Ju alone did not lower any of these factors but a combination of these two significantly induced a lower ratio, meaning less stress on the immune system.

To our surprise, after herbal extract treatment, all the cytokines were increased when mice were treated by a single herb (Figure 5). Obviously, it contradicted the cellular study. One possibility is that in TCM theory, every herb is assigned a profile, which might be hot, neutral, or cold, for treating diseases with these same profiles (Tatsu et al., 2020). However, Gan is neutral while the other herbs in herbal teas all belong to the cold category, which may induce stomach discomfort or inflammation if administrated alone. This could possibly explain why the mice kept losing body weight when fed a single herb (Figures 3A–C).

There have been reports of adverse effect due to ingestion of various herbal teas (Kumana et al., 1983; Sewell et al., 1999; Engels et al., 2013). It has also been indicated that Chinese herbal tea tends to have a negative impact on the spleen and stomach. In the present study, the body weight of mice was monitored after feeding with herbs and it clearly proved that a combination of at least two herbs helped maintain a steady body weight (Figure 3). The importance of Gan is reflected by quantitative formulation with Jin and Ju. Again, our experimental results provided testimony to an old TCM principle on the rationality of formulating different herbs into a one-pot drink.

In TCM theory it is difficult to discriminate the difference of Huo-clearing capability and all so-called Huo-clearing herbs. In the current study, linking inflammation in immunology and ‘Huo’ of TCM enabled comparable quantification of Huo and thus the herbs’ contribution to Huo-clearing could be screened and selected. The rationality of mixing different herbs with a certain ratio was also verified with statistical significance *in vitro* and *in vivo*. The synergy principles for herbal mixtures are common for adaptogens (Panossian et al., 2021) and traditional Indian systems of medicine (Mukherjee et al., 2018).

## Conclusion

There are many kinds of herbal drinks on the market, with various herbs, and different formulas. Although all of these drinks claim a function of Huo-clearing, the lack of an evaluation system makes it difficult for customers to select the correct drink. Our method may aid in future herbs selection and the tea formulations.

## Data Availability

The original contributions presented in the study are included in the article/Supplementary Material, further inquiries can be directed to the corresponding authors.
