# Supplementary material for: Deciphering the Formulation Secret Underlying Chinese Huo-Clearing Herbal Drink
Source: Front Pharmacol. 2021 Apr 22;12:654699. doi: 10.3389/fphar.2021.654699 (PMC8100228; doi:10.3389/fphar.2021.654699)

frontiers

Supplementary Material

Table S1. The quantification of six herbs with chemical standards.

**Herbs**	**Standard compounds**	**RT/min**	**Content (%)**			**Average content (%)**	**SD**	**Standards（r^2 ）**
Glycyrrhiza uralensis	liquiritin	12.3	0.82	0.78	0.82	0.81	0.01341	0.9996
	glycyrrhizic acid	33.7	3.98	2.74	4.17	3.63	0.44867	1.0000
Microcos paniculata	apigenin-8-C-glucoside	31.4	0.03	0.03	0.03	0.03	0.00108	0.9999
Chrysanthemum morifolium	chlorogenic acid	12.2	0.61	0.60	0.63	0.62	0.01001	0.9998
	luteolin-7-O-glucoside	24.8	0.28	0.28	0.27	0.28	0.00325	0.9999
Lonicera japonica	chlorogenic acid	9.6	3.06	4.41	4.04	3.84	0.40487	0.9997
Prunella vulgaris	rosmarinic acid	15.0	0.26	0.43	0.23	0.31	0.06166	0.9998
Plumeria rubra	plumieride	19.6	4.41	3.10	2.42	3.31	0.58354	0.9999

1.1.1

1.1.2 Solution preparation：

The standard stock solutions of liquiritin, glycyrrhizic acid, chlorogenic acid, luteolin-7-O-glucoside, rosmarinic acid, and plumieride were prepared in 70% methanol with a concentration of 200 μg/mL. For sample preparation, lyophilized samples were prepared in 70% methanol with a concentration of 200 μg/mL. The solution was diluted 200 times and then filtered through a membrane filter (0.45 μm) before being injected into the HPLC system for analysis.

Methods for HPLC:

HPLC-DAD analyses were performed using a 1200 Series HPLC-DAD system (Agilent). A C18 column (250 ×4.6 mm, 5.0 µm, Agilent) was used for the chromatographic separations. The mobile phase consisted of 0.1% (v/v) formic acid aqueous solution (A) and acetonitrile (B), using a gradient elution of 5–20% A at 0–8 min, 20–25% A at 8–15 min, 25% A at 15–20 min, 25–40% A at 20–30 min, and 40–60% A at 30–40 min. The wavelength was set at 280 nm. The injection volume was 20 μl, and the flow rate was kept at 1 ml/min.

Figure S1. The quantification of bioactive compounds in six herbs in this study by HPLC.

HPLC of standard apigenin-8-C-glucoside.

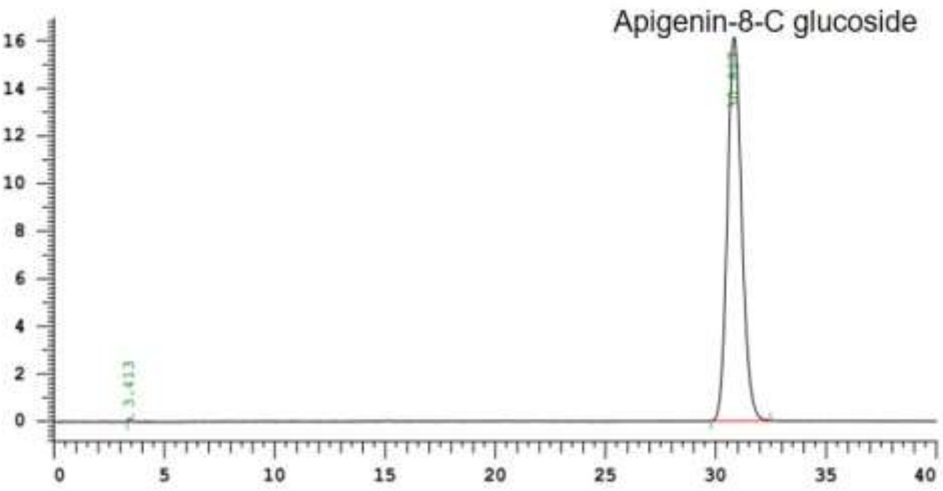

HPLC of apigenin-8-C-glucoside in the herb sample of *Microcos paniculate*.

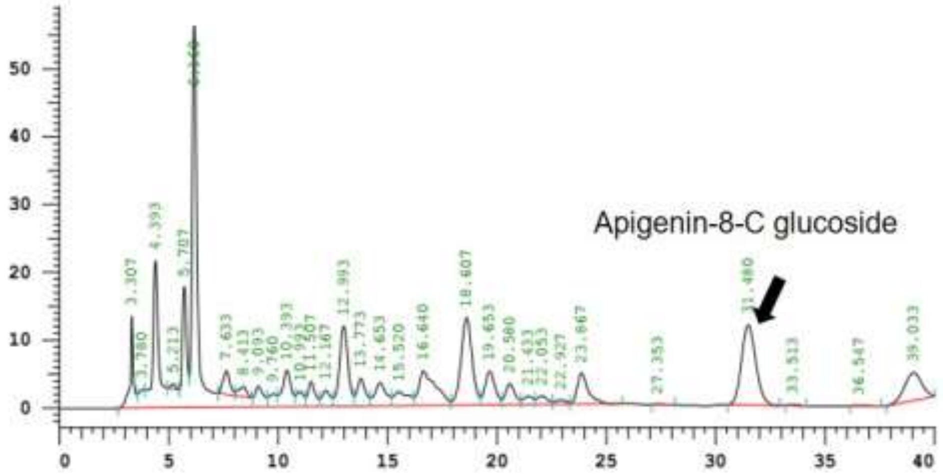

HPLC of standard glycyrrhizic acid.

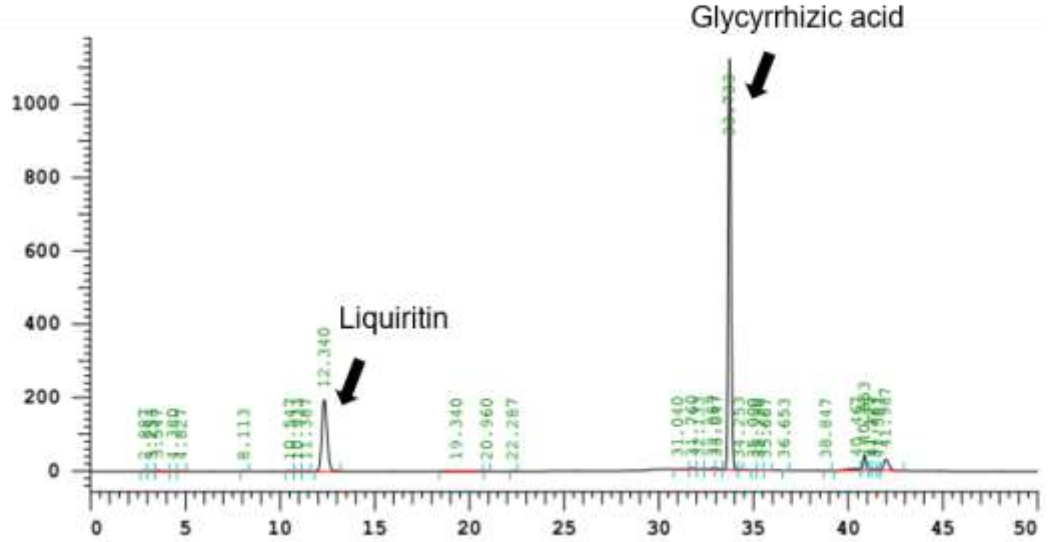

HPLC of glycyrrhizic acid in the herb sample of *Glycyrrhiza uralensis* Fisch.

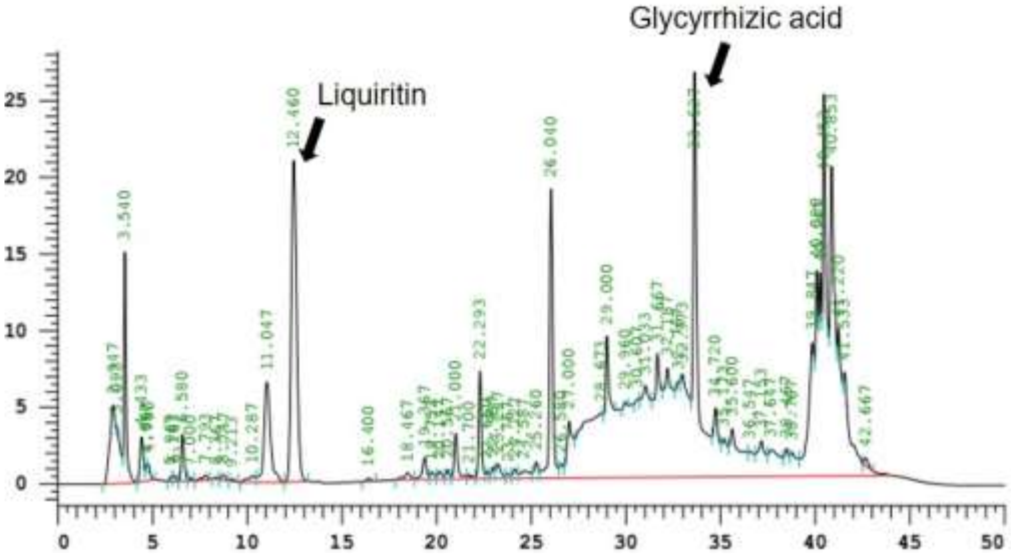

HPLC of standard chlorogenic acid and luteolin-7-O-glucoside.

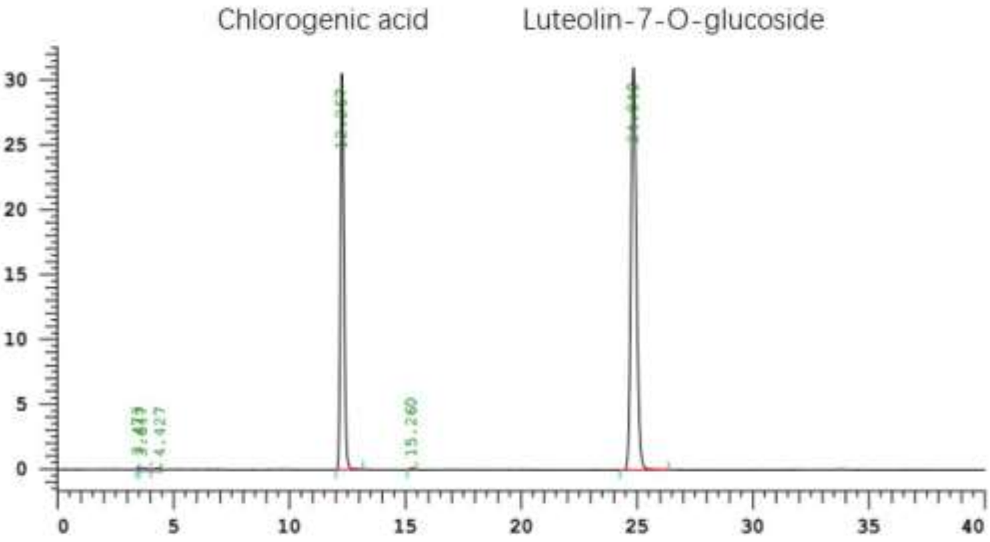

HPLC of chlorogenic acid and luteolin-7-O-glucoside in the herb sample of *Chrysanthemum morifolium* Ramat.

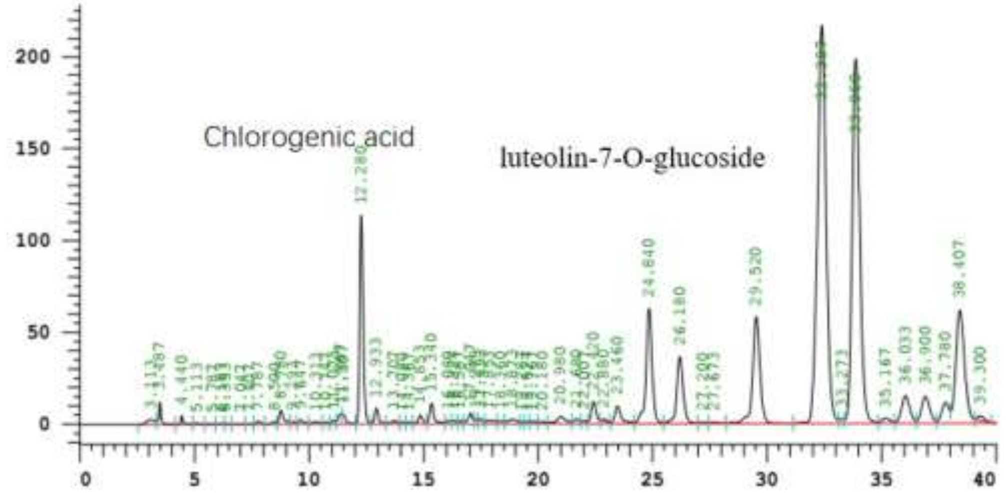

HPLC of chlorogenic acid.

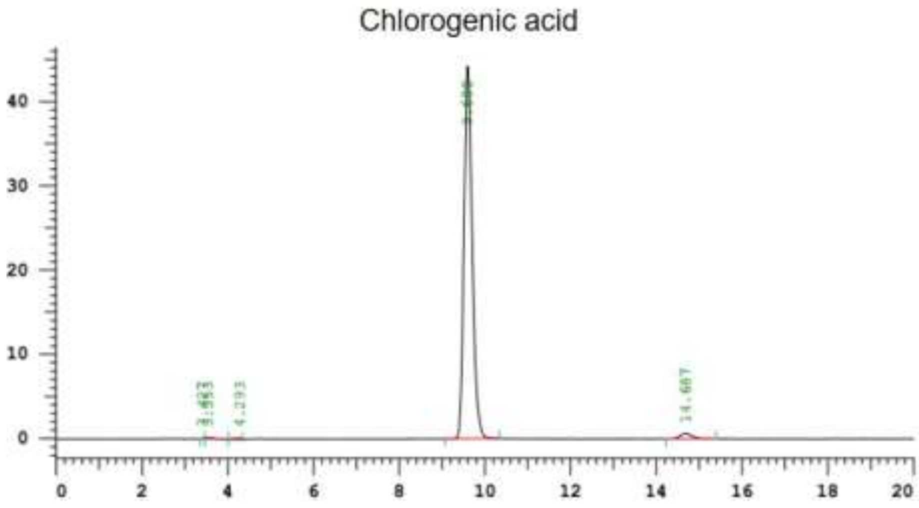

HPLC of chlorogenic acid in *Lonicera japonica* Thunb. herb sample.

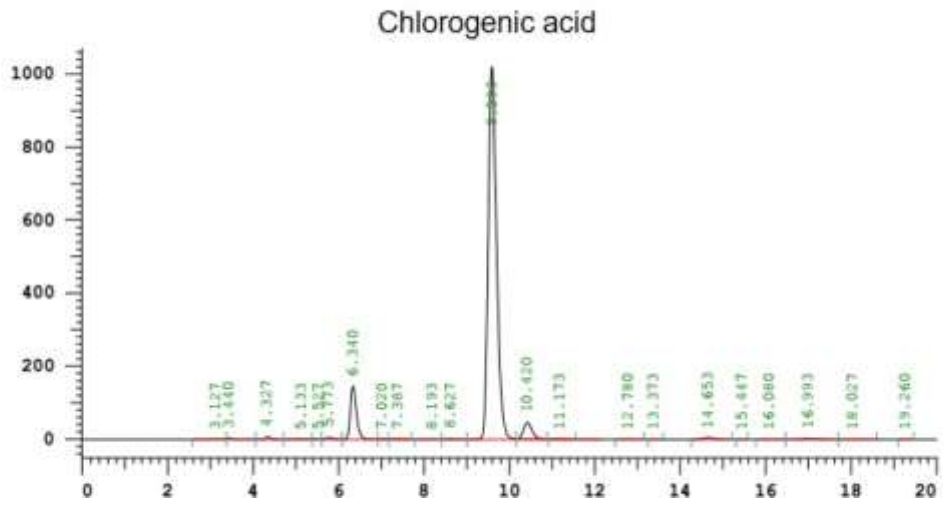

HPLC of standard plumieride.

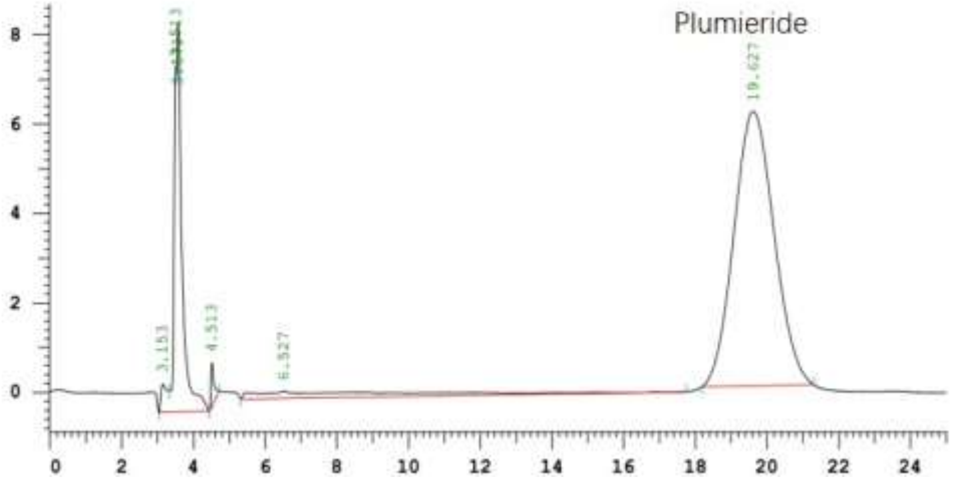

HPLC of plumieride in herb sample of *Plumeria rubra* L.

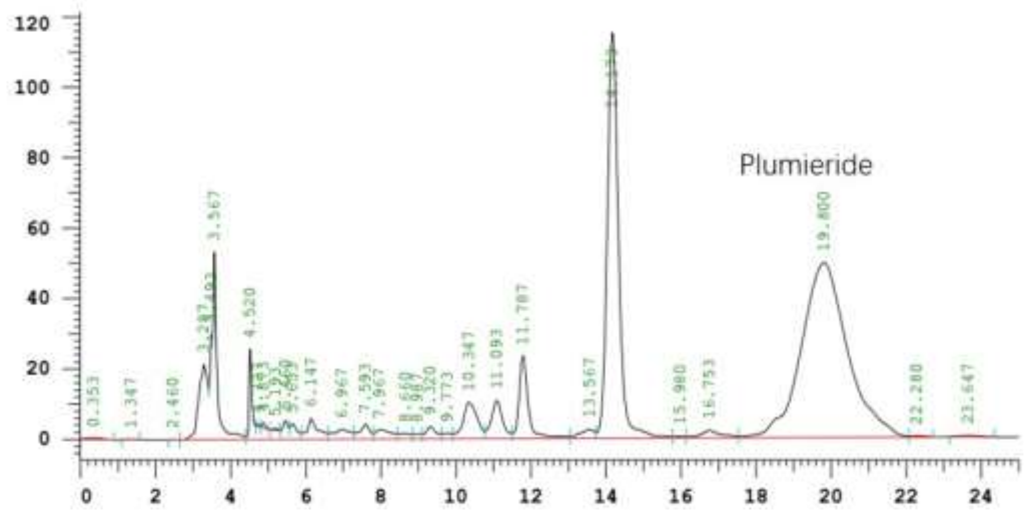

HPLC of standard rosmarinic acid.

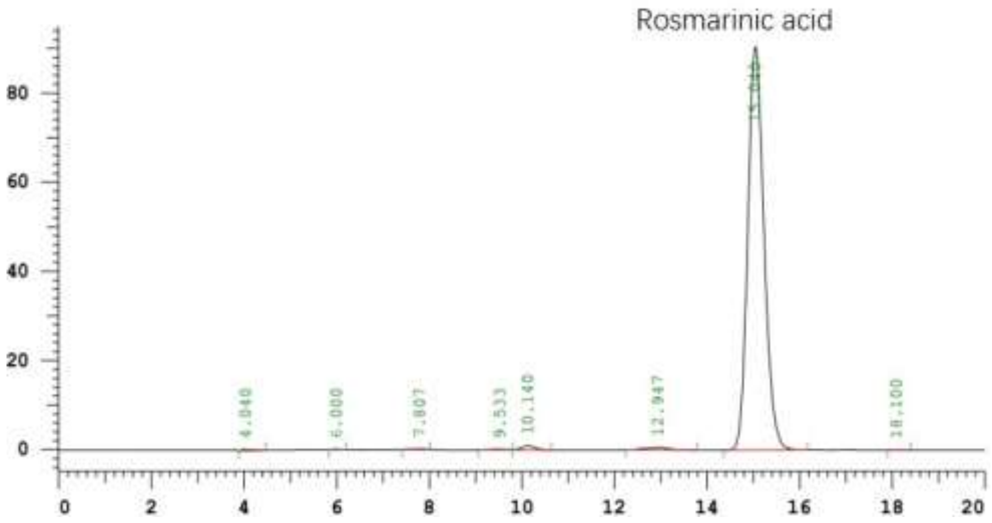

HPLC of rosmarinic acid in herb sample of *Prunella vulgaris* L.

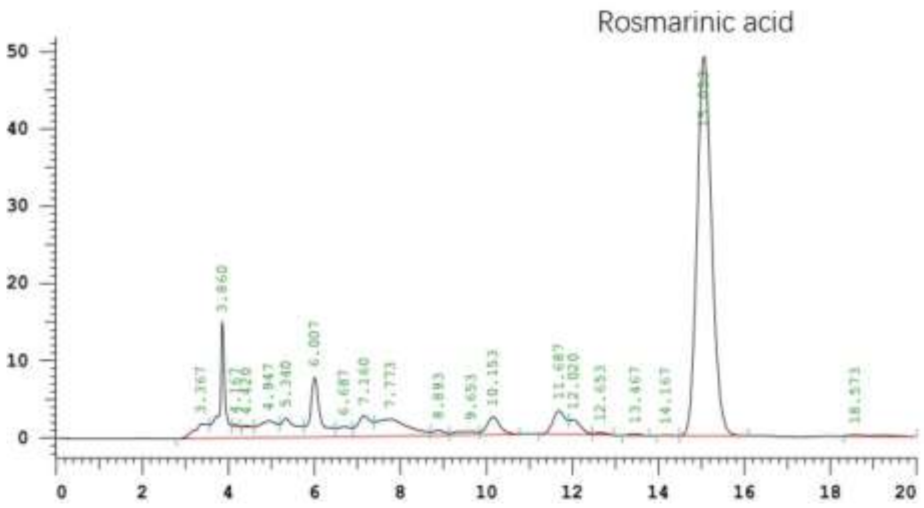

Supplement: Supplementary file 1 [file datasheet1.pdf]
